# malERA: An updated research agenda for combination interventions and modelling in malaria elimination and eradication

**DOI:** 10.1371/journal.pmed.1002453

**Published:** 2017-11-30

**Authors:** The malERA Refresh Consultative Panel on Combination Interventions and Modelling

## Abstract

This paper summarises key advances and priorities since the 2011 presentation of the Malaria Eradication Research Agenda (malERA), with a focus on the combinations of intervention tools and strategies for elimination and their evaluation using modelling approaches. With an increasing number of countries embarking on malaria elimination programmes, national and local decisions to select combinations of tools and deployment strategies directed at malaria elimination must address rapidly changing transmission patterns across diverse geographic areas. However, not all of these approaches can be systematically evaluated in the field. Thus, there is potential for modelling to investigate appropriate ‘packages’ of combined interventions that include various forms of vector control, case management, surveillance, and population-based approaches for different settings, particularly at lower transmission levels. Modelling can help prioritise which intervention packages should be tested in field studies, suggest which intervention package should be used at a particular level or stratum of transmission intensity, estimate the risk of resurgence when scaling down specific interventions after local transmission is interrupted, and evaluate the risk and impact of parasite drug resistance and vector insecticide resistance. However, modelling intervention package deployment against a heterogeneous transmission background is a challenge. Further validation of malaria models should be pursued through an iterative process, whereby field data collected with the deployment of intervention packages is used to refine models and make them progressively more relevant for assessing and predicting elimination outcomes.

## Abstract 2

Richard Steketee and colleagues propose an updated research agenda for combination interventions and modelling in malaria elimination and eradication.

**Summary points**

- Since 2011, there have been significant improvements in the development, organisation, and infrastructure of country programmes for malaria control and elimination globally. This has included the increasing use of combinations of interventions against the mosquito vector and the parasite in humans to reduce transmission in large and expanding geographies and populations and an adaptation of these interventions as transmission is progressively reduced.
- Similarly, there has been substantial improvement in the sophistication and field validation of malaria transmission models and their ability to describe and predict the effects of ecologic changes and the impact of specific interventions. These advances permit the investigation and comparison of multiple complementary interventions in elimination settings.
- There is an increasing need to combine interventions into ‘packages’ that can be tailored to specific settings based on the characteristics of their transmission dynamics and epidemiology (landscape stratification). The challenge is to identify the complementary components of each intervention package and establish the triggers and thresholds for their deployment (or withdrawal) throughout the elimination process, including maintaining elimination once transmission has been interrupted.

## Introduction

In 2011, the Malaria Elimination Research Agenda (malERA) made recommendations for how mathematical modelling efforts could best inform policy and guide research for specific intervention tools for elimination—diagnostics, drugs, vector control, and vaccines [1]. Since then, experience with malaria intervention tools has grown, and the toolbox has expanded with new drugs, new insecticides, better diagnostics, and a first vaccine [2]. As more countries seek elimination, grouping tools to best address diverse and changing transmission intensity has become a central issue. Some tools are oriented primarily towards reducing disease burden, e.g., seasonal malaria chemoprevention; others are dedicated to reducing transmission, e.g., drug-based population-wide parasite clearance; and some meet both of these objectives, e.g., vector control. Thus, not all tools will contribute equally to malaria elimination, and the timing and duration of their use must adapt as programmes progress.

This paper summarises progress since the initial malERA publication regarding transmission-aligned ‘elimination tool packages’ and deployment strategies and opportunities for models to help inform and prioritise intervention choices. The findings come from an extensive literature review of published and unpublished materials and the deliberations of the 2015 malERA Refresh Consultative Panel on Combination Interventions and Modelling, which includes specialists from malaria modelling, field researchers, and National Malarial Control Programme (NMCP) representatives [3].

## Methods

The findings presented in this paper result from an extensive literature review of published and unpublished materials and the deliberations of the 2015 malERA Refresh Consultative Panel on Combination Interventions and Modelling. Electronic databases were systematically searched for published literature from 1 January 2010 until 1 August 2015, without language limitations. The websites of the institutions that apply modelling techniques to malaria research questions and the MESA Track database of current research projects relevant to malaria elimination were systematically searched to identify pertinent ongoing research. Panellists were invited to recommend additional literature and additional ongoing research projects. The comprehensive search for literature and ongoing research provided the basis for launching the second step.

A 2-day workshop was held with the majority of the panel members, including specialists from malaria modelling, field researchers, and NMCP representatives. The panel broke into 2 working groups to identify the issues in combining interventions and how mathematical modelling could be applied to these problems. Each group fed back to a plenary session in which further robust discussions and input occurred. This helped refine the opportunities and gap areas in which research is needed. The final findings were arrived at with inputs from all panellists and several iterations of the manuscript.

## Intervention packages to achieve elimination

**Fig 1:**
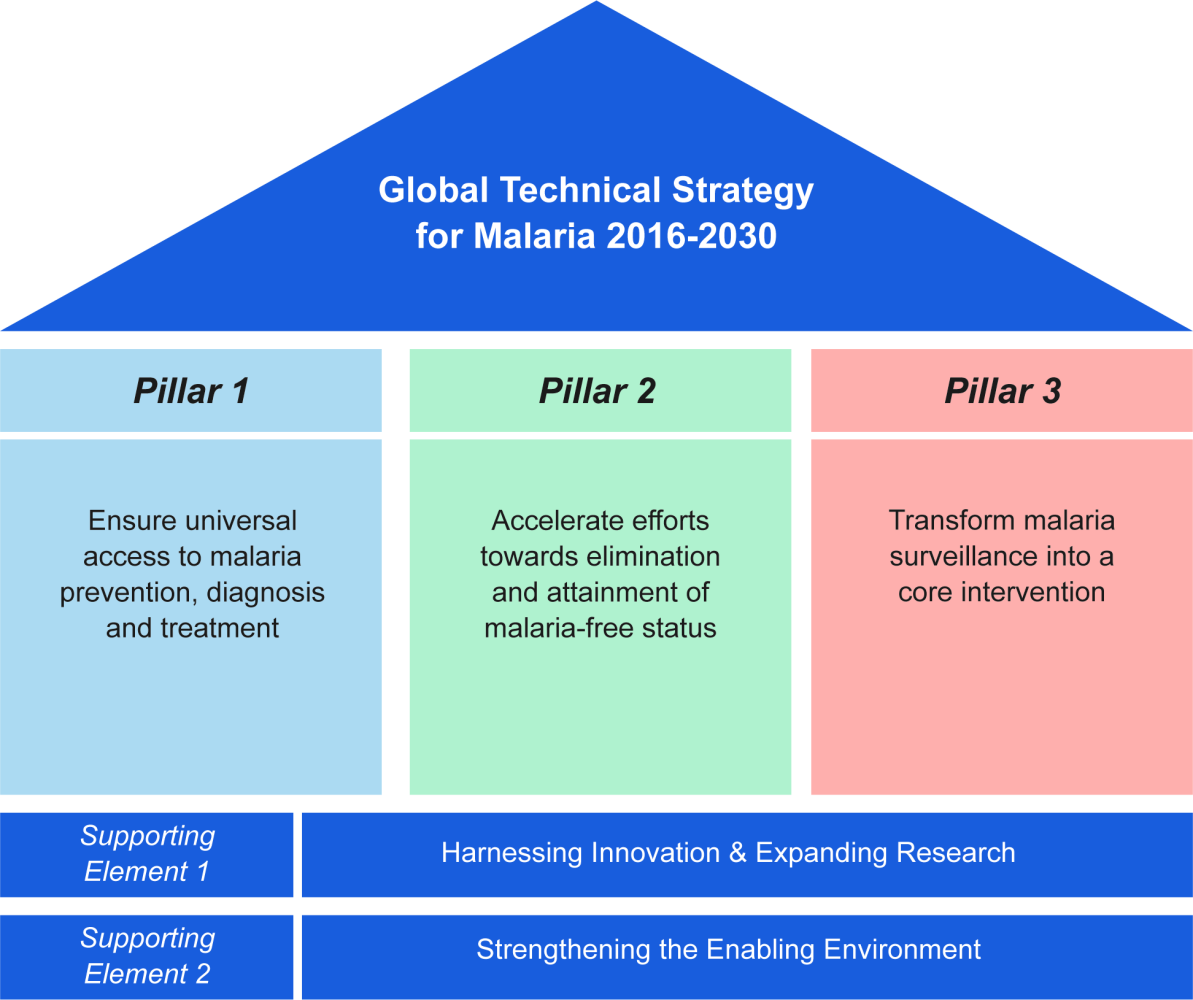
Schematic of the pillars and supporting elements of the World Health Organization (WHO) Global Technical Strategy for Malaria 2016–2030 (source: WHO, 2015) [5].

Over the past 5 years, regardless of initial local transmission levels, most countries have continued to reduce the clinical burden of malaria and transmission [4]. The World Health Organization (WHO) recently published its Global Technical Strategy (GTS) for Malaria 2016–2030 (Fig 1) [5]. This builds on the core activities of vector control, case management, and surveillance, with additional interventions to accelerate progress to elimination. In the GTS, for the first time, modelling studies were used to support goal setting [5].

**Fig 2:**
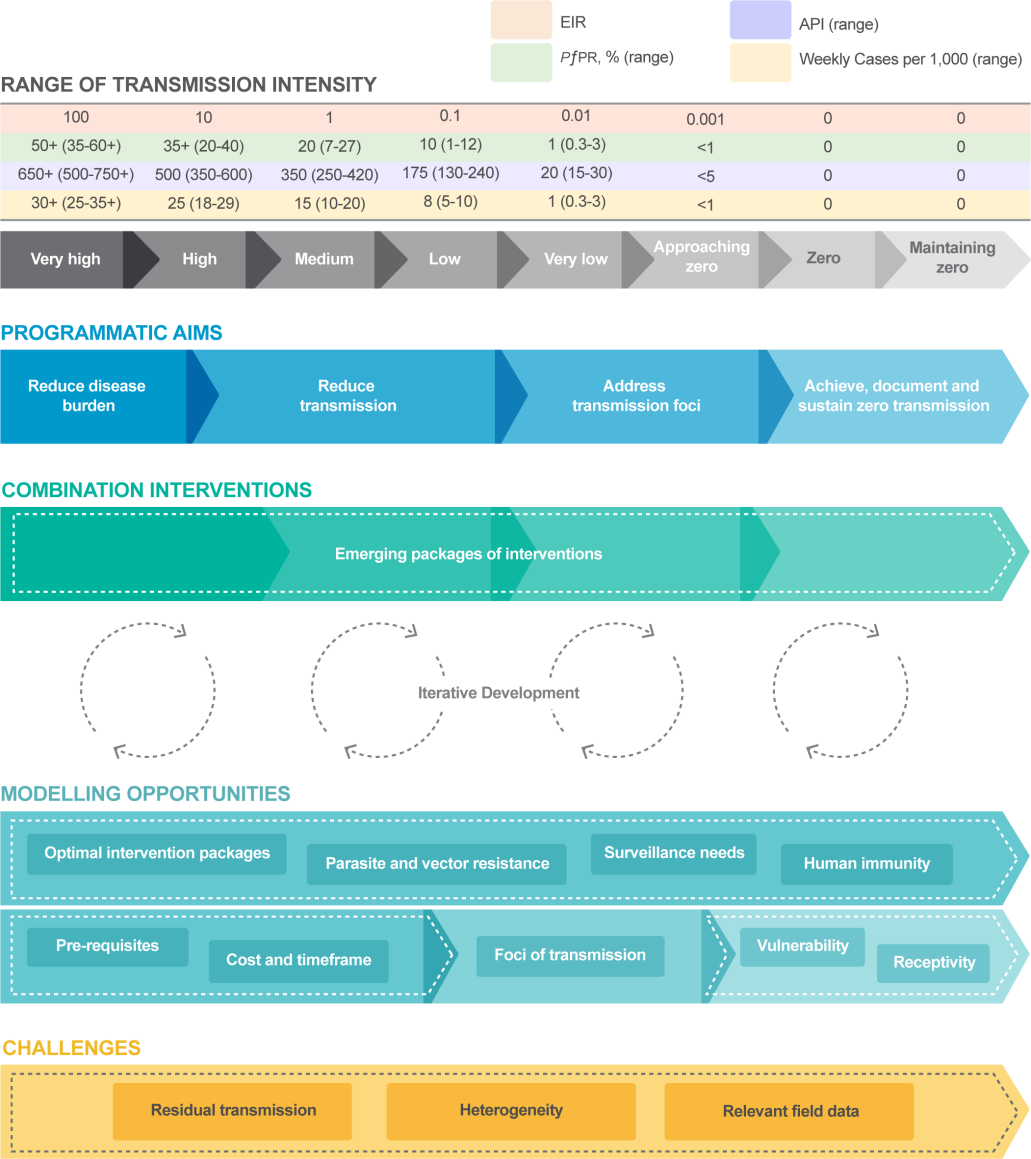
An example of the role of modelling across the spectrum of malaria elimination. Note that the measures of transmission are based on sub-Saharan Africa, and other constructs and transmission levels may be relevant in different geographical areas. Malaria transmission intensity measures and the relationship entomologic inoculation rate for *Plasmodium falciparum* from very high to zero transmission are adapted from data presented in [6]; personal communication from D. Smith and P. Gething. Zero refers to no locally transmitted cases of malaria infection; imported infections may be identified. Intervention package components and sequencing will depend on transmission intensity at the start of the elimination programme, the speed at which transmission declines, and the underlying typology (i.e., malaria epidemiology, species, vector ecology, and health system factors). EIR, entomologic inoculation rate: average number of infectious mosquito bites per person per year; N.B. the table is organised by log differences in the EIR, and other measures are aligned (approximated) based on these entomologic measures. *PfPR*, *P*. *falciparum* parasite rate: proportion of people with a current infection with *P*. *falciparum—*typically determined by a population-based survey and often timed to a specific interval of the transmission season. API, annual parasite index: number of confirmed malaria cases per 1,000 population per year. Cases, cases per health facility per week: average number of confirmed malaria cases expected to present on an average week to a health facility serving a population of 5,000 people. Because many infections can be asymptomatic at any point in time (and thus not present to health services), the proportion of asymptomatic individuals varies with transmission intensity, and because most transmission is seasonal, these average estimates may vary substantially by location and season.

The malERA Refresh Consultative Panel on Combination Interventions and Modelling approach encompassed the full spectrum of malaria transmission—addressing emerging programmatic aims and combining into ‘packages’ the available tools and strategies directed towards malaria elimination (Fig 2). As transmission is reduced to very low levels, the intervention packages must adapt to increasingly focal and heterogeneous populations, in which infections are rare. Given the extensive range of available tools and the diversity/heterogeneity of transmission settings, it becomes difficult to field test all possible intervention packages. Models can assist the prioritisation and design of clinical trials and in the choice of an intervention package to achieve their desired goals.

## Progress in combination interventions and modelling

Initial malERA recommendations for a research and development agenda in mathematical modelling are shown in Box 1 [1]. Subsequently, the scope and depth of research has expanded to include diverse vector control strategies, complex diagnostics, drug and vaccine dynamics, and deployment strategies. Additionally, infection models have advanced following incorporation of new field trial data, particularly regarding mass drug administration (MDA) and specific aspects of vector control, providing greater plausibility to model predictions.

**Box 1. 2011 malERA research agenda for modelling to support malaria elimination.**

Further development of models and model systems:

- Within-host dynamics of *Plasmodium* infections
- The human infectious reservoir
- Bionomics and ecology of the vectors
- Dynamics of the stimulation and decay of human immunity across a range of transmission settings
- Heterogeneities in host, vector, and parasite dynamics
- Heterogeneities in host and vector movements
- Drug pharmacokinetics/pharmacodynamics
- Vaccines that interrupt malaria transmission
- Ecology of genetically modified mosquitoes
- Development and impact of drug and pesticide resistance
- Integration of health system attributes and linking to microeconomic outputs

The interface between modelling and implementation has not developed as was perhaps envisaged, in terms of appropriate portals to allow 'end users' access to relevant software and explore the effect of varying conditions on the ideal choice of control measures. However, the development, organisation, and infrastructure of malaria modelling has improved (Box 2), and recent efforts include an expansion of open-access data and software [6–13]. Also, modelling has been incorporated at the policy level within WHO [5] and included in planning tools for malaria elimination [14]. Wider implementation is possibly now dependent upon the development of next-generation models that sufficiently address combination interventions against a background of heterogeneity and low transmission as more countries move towards elimination.

**Box 2. Recent advances in malaria modelling.**

Communications:

- A growing number of modelling groups are working in a collaborative fashion
- Greater engagement between modellers, country programmes, and operational research partners has helped refine the paramount research questions

Models:

- The development of model systems that are diverse but much improved in terms of their incorporation of malaria biology and natural history, as well as validated estimates for intervention effects, drug pharmacokinetics/ pharmacodynamics, and vaccine dynamics
- The development of models that allow the investigation of target product profiles for new tools—for example, diagnostics, surveillance systems, and drugs

Infrastructure:

- Greater dissemination of malaria models at different levels of user-interface complexity, through online hosting and open-source code repositories leading to wider access to modelling information for programme implementers, planners, and policy decision makers
- Improved means of compiling data and using common ontologies, frameworks, and metadata standards with growing international databases of some measures of malaria transmission, e.g., parasite rate surveys

These advances are complemented by discoveries in basic science, large field trials of new and existing interventions, and substantial data gathering efforts that provide the raw evidence to further validate models. A number of recent reports used models to address the role of multiple complementary interventions (Table 1) [15–35], and additional field trials are ongoing (Table 2) [29].

**Table 1:** Key modelling studies on combination interventions quarter 4 2010–quarter 1 2016, with the main outcome indicated.

**Multi-intervention combined**
• Mass campaigns with antimalarial drugs are highly effective at interrupting transmission if deployed shortly after ITN campaigns [15]. • Compared with untargeted approaches, selective targeting of hot spots with drug campaigns is an ineffective tool for elimination because of limited sensitivity of available field diagnostics [16]. • High coverage with a combination of LLINs and attractive toxic sugar baits could result in substantial reductions in malaria transmission [17]. • Mass treatment needs to be repeated or combined with other interventions for long-term impact in many endemic settings [18]. • Including ivermectin in mass treatment strategies could be a useful adjunct to reduce and interrupt malaria transmission [19]. • Preerythrocytic vaccines will have a maximum impact where bed net coverage has saturated, vector feeding is primarily outdoors, and transmission is moderate to low [20].
**Multi-intervention compared**
• While adult killing methods can be highly effective under many circumstances, other vector control methods are frequently required to fill effective coverage gaps [21]. • Adding vaccines to existing vector control efforts extends the ability to achieve elimination starting from higher baseline transmission levels and with less favourable vector behaviour [22]. • Decreases in malaria transmission and burden can be accelerated over the next 15 years if the coverage of key interventions is increased [23]. • Vector control plans should consider the spatial arrangement of any intervention package to ensure effectiveness is maximised [24]. • The sensitivity of the diagnostic can play a part in increasing the chance of interrupting transmission [25]. • A failing partner drug will result in greater increases in malaria cases and morbidity than would be observed from artemisinin resistance only [26]. • Selecting combinations of interventions that target different stages in the vector's life cycle will result in maximum reductions in mosquito density [27]
**Multi-intervention: Cost-effectiveness**
• In all the transmission settings considered, achieving a minimal level of ITN coverage is a ‘best buy’. At low transmission, MSAT probably is not worth considering. Instead, MSAT may be suitable at medium to high levels of transmission and at moderate ITN coverage [28].

ITN, insecticide-treated bed net; LLIN, long-lasting insecticidal bed net; MSAT, mass screening and treatment.

**Table 2:** Ongoing field studies in combination interventions as reported on the MESA Track database [29].

**Vector control**
• Combining indoor residual spraying and long-lasting insecticidal nets for malaria prevention: a cluster randomised controlled trial in Ethiopia (Maltrials); Ethiopia (Sep 2014–Sep 2016); *Addis Ababa University*, *Ethiopia* • Integrated vector management: Interaction of larval control and indoor residual spraying on *Anopheles gambiae* density and vectorial capacity for human malaria; *Malaria Research and Training Center (MRTC)*, *University of Bamako*, *Mali* • IRS and LLIN: Integration of methods and insecticide mode of actions for control of African malaria vector mosquitoes; Tanzania, United Republic of; *Ifakara Health Institute (IHI)*,* Swiss Tropical and Public Health Institute (Swiss TPH)* • Cluster randomised trial of the impact of dual-insecticide treated nets vs. traditional LLINs on malaria vectors and malaria epidemiology in 2 districts of Mali; Mali (Dec 2013–Dec 2014); *Centers for Disease Control and Prevention (CDC)*, *United States* • The Majete Integrated Malaria Control Project (MMP): Community-based malaria control in the perimeter of Majete Wildlife Reserve in Chikhwawa district using a Scale-Up-For-Impact (SUFI) strategy, assessing complementary intervention options, including larval source management and house improvement; Malawi (Jan 2014–Dec 2018); *Wageningen University*, *Netherlands; University of Amsterdam; College of medicine*, *University of Malawi; Liverpool School of Tropical Medicine*
**Case management and surveillance**
• Routine case investigation and reactive case detection for malaria elimination in Richard-Toll District in northern Senegal; Senegal (2012–2017); *PATH MACEPA*,* National Malaria Control Programme (NMCP) Senegal*
**Mass treatment**
• The Haiti Malaria Elimination Consortium (HaMEC); Dominican Republic, Haiti (Feb 2015–2020); *Malaria Zero Consortium*, *US* • Assessing the effectiveness of household-level focal mass drug administration and community-wide mass drug administration with dihydroartemisinin + piperaquine for reducing malaria parasite infection prevalence and incidence in Southern Province Zambia; Zambia (2014–2016); *PATH MACEPA*,* Tulane University*, *Zambian National Malaria Control Centre* • Population parasite clearance to decrease malaria transmission in Amhara Region, Ethiopia: a pilot study; Ethiopia (2014–2015); *PATH MACEPA*,* Ministry of Health (MOH) Ethiopia* • Reduction of malaria parasitaemia and transmission in low to moderate seasonal transmission settings (Kanel, Ranérou and Linguère) in Senegal: a pilot study; Senegal (2014–2015); PATH MACEPA, National Malaria Control Programme (NMCP) Senegal • Community reactive case detection versus reactive drug administration in malaria elimination areas: a cluster randomised controlled trial; Zambia (2016–Dec 2017); *Akros* • Assess the micro-epidemiology of resistant falciparum malaria in SE Asia and to perform and evaluate an intervention with targeted chemo-elimination through a modified mass drug administration approach (Cambodia, Myanmar, Thailand, Vietnam); Cambodia, Myanmar, Thailand, Vietnam (2014–Oct 2016); *Mahidol Oxford Tropical Medicine Research Unit (MORU)* • Evaluation of the impact of seasonal malaria chemoprevention delivered by district health services in southern Senegal; Senegal (2013–2018); *Cheikh Anta Diop University*, *Senegal*

IRS, indoor residual spraying; LLIN, long-lasting insecticidal net; SE, Southeast.

### Consensus modelling

In consensus modelling, independent modelling groups examine the same research question, sometimes using the same source dataset to parameterise their model. Through objective comparison and critique, modelling groups have reached a degree of consensus on important issues, such as the relationship between health burden and transmission intensity [6], and have undertaken an in-depth analysis for the RTS,S vaccine [36]. Such efforts are resource intensive but may give robust answers incorporating the breadth of uncertainty in our understanding. There is also value in less intensive forms of model comparison in which common findings from work conducted independently are assessed (Table 3) [9,18,21,23,24,28,30–32,35,37–58]. This approach can also be particularly useful for identifying areas in which there is a lack of consensus, as this can focus efforts on further model development, basic science, and field data collection needs.

**Table 3:** Consensus across multiple groups from modelling analyses conducted by each of the Malaria Modelling Consortium^a^ groups, which assessed impact on malaria transmission of combining multiple interventions or multiple methods of using a single intervention^b^.

**Vector control**
• Achieving and maintaining high effective coverage of the population with LLINs is consistently predicted to result in the greatest reduction in transmission in a variety of settings and in many cases enables other interventions to become more effective and longer lasting [21,23,24,28,30,32,35,37–43,55]. • Other interventions such as IRS are also predicted to be effective and can even be more effective than LLINs in specific settings, particularly if sustained and optimised through seasonal or spatial targeting strategies [32,39,42]. • Vector control interventions that maximise killing of adult female mosquitoes are predicted to have the greatest transmission reducing effect (as opposed to repellents or killing juveniles); however, the optimal choice of intervention(s) will depend on both the specific bionomics of local vectors and the costs required to reach high levels of effective coverage with each intervention [21,23,44–46].
**Case management and surveillance**
• Even before considering elimination, improving access to care has an important role to play in significantly reducing deaths and severe disease [9,41,47–49]. • While differing considerably in magnitude, all the models agree that levels of access to treatment of incident malaria cases and the delay in seeking treatment are 2 key measures that influence the endemicity at baseline (no interventions) and, as such, determine the following: ○ what scale of community-based programme will be required to achieve and maintain elimination [28,30,32] ○ what the risk will be of scaling back vector-based interventions post elimination [23,43,50,51]
**Mass Treatment**
• Short mass treatment campaigns will reduce the parasite reservoir—and consequently, transmission—in the short term but will have no long-term benefits unless other interventions are scaled up at the same time and then maintained [18,23,28,31,32,35,42,52–55]. • Treating a large proportion of the population in a single year in at least 1 round is a key determinant of MDA effectiveness whether it is achieved through high coverage in a single round or through follow-up rounds that reach new individuals [41,53,55–57] • The addition of primaquine to MDA with long-lasting ACTs offers a small additional transmission reduction in the majority of epidemiological settings [18,30–32,42,53,54,57,58]. • Due to the prophylactic effect of treatment, MDA will always be more effective than MSAT or fMDA. If adherence or drug resistance is included in the model analysis, then this conclusion is more nuanced, and risk of drug resistance emergence and spread is an area with a lack of clear consensus among existing models [18,31,35,41]. • The longer-term effectiveness of MDA is highly sensitive to the population size of the trial area and its connectedness to other areas [18].

^a^ Imperial College, London, United Kingdom; Institute for Disease Modelling, Seattle, Washington, US; Mahidol Oxford Tropical Medicine Research Unit, Bangkok, Thailand; Swiss Tropical and Public Health Institute, Basel, Switzerland; and University of Oxford, Oxford, UK.

^b^ Compiled by Oliver Brady (University of Oxford) and Samantha Galvin (Bill & Melinda Gates Foundation).

ACT, artemisinin-based combination therapy; fMDA, focal mass drug administration; IRS, indoor residual spraying; LLIN, long-lasting insecticidal bed net; MDA, mass drug administration; MSAT, mass screening and treatment.

## Next steps for combination interventions and modelling in malaria elimination

Fig 2 provides an example of how transmission strata, programmatic aims, the choices of intervention packages, and the iterative development between modelling and programme choices change together as malaria transmission intensity is progressively reduced towards zero, summarizing key opportunities and identifying challenges. Note that not all countries will start from high transmission levels and that the measures of transmission used in Fig 2 are based on sub-Saharan Africa. Thus, other constructs and transmission levels may be relevant in different geographical areas.

## Opportunities

### Combination intervention modelling

There has been considerable progress in modelling combination interventions. Models have been developed to examine the overall expected impact of diagnostic, drug, vaccine, and vector control intervention combinations, including cost-effectiveness [16,18–20,24,48,57,59–62], and comparing interventions added to the backbone of standard measures [21–23,25–27,30,36,63,64]. Modelling studies have investigated the applications of several new potential interventions such as the RTS,S vaccine [36], ivermectin [19,54], mosquito traps [17], and next-generation diagnostics [25,33,65,66] and have highlighted critical attributes of new products, such as a preerythrocytic vaccine [20,67–69], genetically modified mosquitos [70–72], and combinations of future interventions [73].

Models are designed to allow scale-up and scale-down of interventions over time. The next step is to define the epidemiological information that would be most informative for making such dynamic changes and the triggers for switching or scaling. The aim is to develop a set of rules that define the characteristics of transmission that can direct specific changes in the composition and phasing of intervention packages and their targeting to specific locations and populations. These predictions can then be evaluated with further evidence from specific field trials. If reliable, such measures could be used in the subnational stratification of intervention packages.

#### Accelerating community clearance of malaria parasites

One hypothesis being tested in various settings is the potential to accelerate elimination by targeting the human parasite reservoir (symptomatic and asymptomatic) with time-limited deployment of community-based interventions such as MDA or mass screening and treatment (MSAT) [74]. If the intervention is justified, a wealth of modelling studies provides guidance on optimizing its deployment [15,18,28,32,33,41,53–55,57,75–79]. However, estimating the level of coverage required for successful MDA is critical [53], and for MSAT, the sensitivity of the diagnostic tool is an additional key determinant of efficacy as the current tests may fail to detect low-level infections [16,25].

Current models of MDA all include the parameters whereby immediately following MDA, there is a dramatic drop in malaria prevalence, but in the absence of elimination, prevalence returns to preintervention levels (albeit at different rates depending on the model) [53]. Country malaria programs are increasingly aware of this potential and have learned not to rely solely on MDA to eliminate transmission; thus, MDA is an accelerator used to move to a next set of interventions and strategies to find and clear the remaining transmission foci. The models must now be adapted to include a next set of actions with the potential to end transmission, i.e., MDA moving to focal MDA (fMDA) and other reactive strategies in households and neighbourhoods with rare but remaining transmission [33,79,80]. In the field, these increasingly infrequent actions will require robust local information systems as part of the intervention, rather than models.

#### Non-falciparum species

Recent progress has been made in models considering non-falciparum parasites and vectors, though further work is needed [76,81–95]. To address the public health and public engagement challenge of eliminating all human malaria species, multispecies mathematical models that consider unified strategies and exploit the interactions between the species for improved cost-effectiveness should be used [96]. Notably, where *P*. *vivax* is present, the malaria programme might be sustained even as *P*. *falciparum* becomes rare and is eliminated. However, different approaches to both surveillance and malaria interventions would be required to reduce the *P*. *vivax* burden while detecting *P*. *falciparum* cases and preventing the reestablishment of *P*. *falciparum* transmission.

### Surveillance as an intervention

Surveillance is an intervention tool. When honed for elimination purposes, surveillance must evolve to be able to discover evidence of transmission; establish its location, timing, nature, and causes; identify and eliminate residual foci; prevent, detect, and contain imported malaria; and demonstrate the attainment and maintenance of zero malaria transmission [97]. As transmission declines, modification of data collection and reporting systems requires substantial investment and coordination across the malaria programmes and the surveillance management unit. Designing the necessary flexibility into a surveillance system to allow for adaptation to an elimination context will be critical.

There is an opportunity to use modelling to define the required components of surveillance systems depending on the stage of the elimination programme. This requires quantification of the detrimental effects of inaccurate, insufficient, or untimely surveillance and the beneficial effects of adding new measures to the surveillance system [25]. Modelling could also be used to assess the level of hidden/unidentifiable cases/infections that would hinder (or would not hinder) elimination (e.g., asymptomatic or individuals with minor symptomology who would not seek treatment). As transmission declines, the addition of serological measures of past exposure [65,98–102] or active community-based transmission measurements and reactive case management [103–107] may be considered. Modelling can estimate the incremental benefit of adding specific surveillance activities to an already established surveillance system and could examine cost-effectiveness issues [48,108], specific epidemiologic aspects of contract tracing [109], and the target product profile of diagnostics [25,65,66] in case-investigation or foci-investigation settings.

### Parasite and vector resistance

As efforts to reduce transmission are intensified, the risk and impact of parasite drug resistance and vector insecticide resistance becomes a key concern [110–112]. Modelling has been used to investigate the effects of resistance [25,26,30,32,113–116], and there have been some studies examining risk factors for resistance and drug failure [114,117–119]. Geostatistical models are also being developed to predict localities where resistance might be present in order to target surveillance activities, for example, mapping artemisinin-resistance in Southeast Asia [120]. The biology and natural history of mosquito vectors and malaria parasites tells us that the development and evolution of resistance will continue, given the pressure of insecticides and drugs. In terms of drug treatments, with artemisinin-based combination therapies (ACTs) globally recommended for malaria treatment, the focus must be on investigation of artemisinin and partner drug resistance, in terms of how this can be contained within the Greater Mekong subregion [111], and how its emergence or importation can be avoided in other regions [25,115]. Note that as transmission declines, the remaining parasites are those most likely to harbour resistance. Thus, even as malaria cases decline, continued field studies and modelling must be supported to address the efficacy and effectiveness of intervention tools critical for elimination programming. The next steps are to investigate how packages of interventions can be modified to mitigate the effects of resistance on existing interventions [30,121–123], how resistance can be contained [32], and how resistance can be avoided, particularly for new drugs and insecticides [124,125].

### Human immunity

A gradual decline in human immunity to malaria across the population is an inevitable consequence of reducing malaria transmission and contracting parasite diversity [126,127]. The resulting delay in acquiring immunity likely will alter the age distribution and severity of malaria infections [126,128,129]. Understanding these changes is necessary to identify the most vulnerable populations or those most likely to need an intervention [128,130]. Models already include age-dependent immune factors and have dynamic modulation of immunity as a function of entomological inoculation rate [128,131], though additional temporal data could help reduce the uncertainty surrounding these functions. Gaps remain in our understanding of immunity in areas of long-standing low transmission (e.g., Haiti), where the level of asymptomatic infections is much higher than previously thought [132].

### Modelling to inform policy

Strategic decisions are already being taken as part of elimination planning in a number of countries. There are numerous opportunities for modelling to inform these decisions—for example, scenario planning. An Elimination Scenario Planning (ESP) toolkit was published by WHO in 2014 following field testing using data from The Gambia and Senegal [14]. The manual is linked to software that models malaria transmission (currently limited to *P*. *falciparum* in Africa), which allows users to explore the effect of a range of combinations of malaria control interventions in order to achieve elimination. Such an approach has wide application and could be extended to *P*. *falciparum* outside Africa or *P*. *vivax* settings in the future. A key consideration is that malaria policy will need to respond to climate change. Historical data may become less reliable as seasonal patterns of rainfall and land use alter. Mapping climate change effects and possible scenarios following the varied consequences of climate change for human and vector population distributions has been investigated at continental and national levels, but incorporating this into policy is more challenging [133–151].

Mathematical models can provide a framework for exploring the relationship between population movement, heterogeneous transmission, and the deployment logistics of a national or regional elimination strategy. To carry out such analyses, new model frameworks should be developed that benefit from new field and genetic data characterising and measuring spatially and temporally dynamic transmission routes.

There is an increasing demand from NMCPs for pertinent and prompt mathematical modelling analyses to support their malaria elimination strategies. Established modelling groups have engaged in local capacity building. Also, malaria modelling research is being published by research groups from malaria-endemic countries [33,34,62,89,152–154], and this trend could be supported to the benefit of NMCPs.

### Modelling to maintain zero

As noted above, when transmission becomes rare, models are increasingly challenged in informing policy and intervention choices; similarly, when there is no transmission, the evaluation of risk for the reintroduction of infection (vulnerability) and the risk of propagating local transmission given its reintroduction (receptivity) can present challenges to models designed to answer questions at high endemicity levels. A new class of highly heterogeneous, stochastic malaria models is being developed to inform the design of an elimination surveillance system.

#### Vulnerability (risk of introduction or reintroduction)

Measuring vulnerability to malaria reintroduction requires pairing up-to-date maps of national and international parasite prevalence with human movement models. Both of these fields have advanced in recent years [33,34,117,155–160]. Human movement models, paired with travel survey and microcensus data, have improved their description of routine human movement (e.g., holiday season travel) [159,161]. Increasing use of mobile phones has enabled the tracking of human movement and permitted distribution advice on infection avoidance [159,162–164]. However, many national and international seasonal migrations remain difficult to predict, and their direct relationship to moving malaria infections requires additional investigation.

#### Receptivity (risk of transmission given introduction)

In order to direct interventions, models must incorporate both the risk of importation and the risk for the reestablishment of local transmission [165–173]. The risk of malaria transmission reestablishment can be measured as a function of selected host, vector, and environmental data [156,170,171, 174]. For example, measures might include human use of insecticide-treated bed nets or indoor residual spraying, mosquito habitat suitability and its link to abundance, and climatic conditions (e.g., temperature, rainfall, and vegetation index measures) that support or accelerate vector and parasite development. If such data are collected widely enough, models can be validated using the occasional areas that do experience local transmission. Deciding which environmental and entomological data would be most valuable to collect could be iteratively informed by testing hypotheses based on longitudinal data from areas that have recently eliminated malaria, for example, Sri Lanka. The next step is to translate risk mapping into programmatic actions, such as better allocation of human resources, and maintenance and targeting of vector control [50,175]. This will become increasingly important as more countries reach elimination.

## Challenges

### Residual transmission

Variable human and vector behaviours may enable sustained transmission in highly seasonal, heterogeneous environments, despite high intervention coverage [176]. The magnitude and importance of residual transmission in different settings require further field studies. In particular, human sociobehavioural data including human behaviour’s relevance for compliance and entomological data investigating the contribution of outdoor transmission are needed to develop models testing novel strategies and tools [102].

### Low transmission and incorporating heterogeneity

Models have mostly been used to examine sub-Saharan Africa high transmission contexts with *P*. *falciparum* and relevant vector species, though they may be parameterised across the full spectrum of transmission. When modelling an isolated homogeneous population, it can be difficult to sustain transmission much below the 1% parasite prevalence level (though the precise level depends on the model), with the model becoming unstable, leading to ‘stochastic extinction’, i.e., the extinction of parasites based on random effects within the model, an effect that is compounded with increasing heterogeneity [177]. This suggests that importation of infections and local heterogeneities in host, vector, and parasite dynamics and in health service delivery systems are likely to play an important role in sustaining malaria in low transmission settings [178].

As a country progresses to very low levels of malaria transmission, the spatial and temporal heterogeneity of transmission increases in importance. In these contexts of varying historical transmission intensity, intervention coverage, human movement, and access to health system resources, malaria will tend to persist in the most remote regions and the poorest and most vulnerable populations [179,180]. While this issue may not require new models per se, heterogeneity will need to be better captured as transmission declines. Spatial heterogeneity is probably least well developed, and the required level of spatial granularity and relevant metrics for answering specific questions in low transmission settings requires definition [181,182]. However, at some point heterogeneity will exceed the ability of models to establish granularity, and decision making will require local health system and entomological data.

### Modelling malaria at borders

When malaria transmission is moderate to high and similar on both sides of a border, often little attention is paid to border areas for specific disease interventions; however, this changes when one nation may be markedly reducing transmission and the other is not. Border areas present particular difficulties for malaria control and elimination efforts [183–187]. The complexity of human movements for trade, business, and visiting family, sometimes including vulnerable populations [188], and the coordination of efforts between different political and organisational frameworks increase the complexity of malaria control [184]. Some of the issues relate to spatial and temporal heterogeneity and could possibly be addressed with greater data on human cross-border movement and parasite genetics [189–191]. However, human factors, such as local conflicts, poverty, and the disenfranchisement of particular ethnic groups, can be highly variable in time and place and are more challenging to incorporate into transmission models [192,193]. Alternative complementary approaches include mapping malaria risk, for better targeting of resources, plus goal setting by modelling what could potentially be achieved with coordinated versus independent elimination campaigns [33,185,194,195]. Once the potential benefits are understood, the barriers to reaching these goals can be researched and the feasibility of overcoming them explored.

### Iteration and validation

Finally, models directed at assessing combination interventions must embrace a process of iteration with field data. In particular, data are needed from low to near-zero transmission settings. Such data needs might include high-resolution geographic information on cases, frequency and location of associated secondary cases, travel history identifying infection sources, vector-associated data, climate, and environmental parameters [109]. The requirement for field data to validate models remains problematic, as field data on intervention efficacy and the diverse parameters noted above can be difficult to assemble. When developing models, validation requirements should be clearly defined and data should be feasible to obtain. Amidst these challenges, modellers then need to consider how to best contribute to and bear responsibility for the assembly of required field data. Although capacity building and integration of modellers into NMCPs may address this at a local scale, there is a need for innovative mechanisms to allow increased exchanges in malaria elimination research, to allow better access to field empirical data for modellers.

## Conclusions

Given the ongoing social and economic impact of malaria-related mortality and morbidity and the inevitable resource constraints for national malaria programmes, identifying the most timely and most cost-effective path to malaria elimination is a priority. Box 3 presents a research and development agenda for combination interventions and modelling in malaria elimination. Modelling affords a feasible and practical means of investigating rational combinations of interventions and the most appropriate setting for their deployment. Nevertheless, without a substantive dataset from operations research, the construction of meaningful models is not possible. Models must also be continuously validated against field data, through programmatic experience and against clinical trials, with measures and outcomes data relevant to the transmission setting identified and collected for use in further model refinement. This is especially the case as we increasingly encounter transmission settings that are shrinking in size and number and becoming more focal and heterogeneous and for which there are fewer field data. Thus, there is a codependency between modelling and field data, and the quality of both must be assured for findings to be valid and impactful. Since malERA 2011, there has been significant progress in aligning modelling with programmatic requirements and more effective communication with policy makers. This ongoing dialogue will ultimately determine the relevance of modelling to policy decision and its contribution towards achieving and maintaining malaria elimination.

**Box 3. Research and development agenda for combination interventions and modelling.**

- Determine which combinations of interventions to use in which sequence and in response to which triggers throughout elimination
- Identify the circumstances in which time-limited elimination acceleration interventions, such as mass drug administration (MDA), are appropriate and what needs to be done to retain the gains in transmission reduction following their withdrawal
- Model the effect of parasite drug and vector insecticide resistance on combination interventions and how resistance might be avoided or contained
- Understand human immunity in areas where transmission has always been low and parasite diversity very low and modelling the effect of changes in human immunity as transmission declines
- Identify which additional data would be most useful for validating or changing model predictions in order to drive iterative development and decision making

**Surveillance** **as an intervention**

- Model the target product profile of an elimination-specific surveillance system
- Determine the threshold at which reactive case strategies become feasible

**Strategic** **modelling**

- Estimate the long-term costs of elimination in different settings and with different intervention packages
- Assess the potential duration of an elimination campaign in various settings to help define the investment case and financing needs for elimination
- Estimate the maximal impact of currently available tools on elimination in various settings
- Determine the counterfactual to elimination, i.e., the effect of continuing current interventions in various settings
- Support capacity building of modellers embedded in National Malaria Control Programmes (NMCPs)

**Modelling** **to maintain zero**

- Investigate how vulnerability and receptivity measures can be translated into specific programme actions

**Addressing** **transmission**

- Apply models to low transmission settings, incorporating all relevant parasites/vectors
- Investigate the importance of residual transmission in different settings and what new strategies or novel tools are needed to overcome it

**Incorporating** **heterogeneity**

- Determine the relevance of spatial and temporal heterogeneity in transmission in different settings
- Investigate how much heterogeneity in transmission needs to be captured by models to make predictions in elimination settings

**Iteration** **and validation**

- Determine which measures of transmission or other metrics are most appropriate for guiding programmatic decisions in low transmission to maintaining-zero settings
- Define which new data need to be collected from low transmission to maintaining-zero settings in order to increase confidence in model predictions

## Abbreviations

ACT: artemisinin-based combination therapy
ESP: Elimination Scenario Planning
fMDA: focal mass drug administration
IRS: indoor residual spraying
LLIN: long-lasting insecticidal bed net
malERA: Malaria Eradication Research Agenda
MDA: mass drug administration
MSAT: mass screening and treatment
NMCP: National Malarial Control Programme
WHO: World Health Organization
